# Auditory Selective Attention Reveals Preparatory Activity in Different Cortical Regions for Selection Based on Source Location and Source Pitch

**DOI:** 10.3389/fnins.2012.00190

**Published:** 2013-01-07

**Authors:** Adrian K. C. Lee, Siddharth Rajaram, Jing Xia, Hari Bharadwaj, Eric Larson, Matti S. Hämäläinen, Barbara G. Shinn-Cunningham

**Affiliations:** ^1^Athinoula A. Martinos Center for Biomedical Imaging, Massachusetts General HospitalCharlestown, MA, USA; ^2^Department of Speech and Hearing Sciences, Institute for Learning and Brain Sciences, University of WashingtonSeattle, WA, USA; ^3^Center for Computational Neuroscience and Neural Technology, Boston UniversityBoston, MA, USA; ^4^Department of Biomedical Engineering, Boston UniversityBoston, MA, USA

**Keywords:** frontal eye fields, superior temporal sulcus, magnetoencephalography, auditory attention, auditory spatial processing, pitch processing

## Abstract

In order to extract information in a rich environment, we focus on different features that allow us to direct attention to whatever source is of interest. The cortical network deployed during spatial attention, especially in vision, is well characterized. For example, visuospatial attention engages a frontoparietal network including the frontal eye fields (FEFs), which modulate activity in visual sensory areas to enhance the representation of an attended visual object. However, relatively little is known about the neural circuitry controlling attention directed to non-spatial features, or to auditory objects or features (either spatial or non-spatial). Here, using combined magnetoencephalography (MEG) and anatomical information obtained from MRI, we contrasted cortical activity when observers attended to different auditory features given the same acoustic mixture of two simultaneous spoken digits. Leveraging the fine temporal resolution of MEG, we establish that activity in left FEF is enhanced both prior to and throughout the auditory stimulus when listeners direct auditory attention to target location compared to when they focus on target pitch. In contrast, activity in the left posterior superior temporal sulcus (STS), a region previously associated with auditory pitch categorization, is greater when listeners direct attention to target pitch rather than target location. This differential enhancement is only significant after observers are instructed which cue to attend, but before the acoustic stimuli begin. We therefore argue that left FEF participates more strongly in directing auditory spatial attention, while the left STS aids auditory object selection based on the non-spatial acoustic feature of pitch.

## Introduction

The ability to selectively attend to one of multiple simultaneous sensory stimuli is very flexible, allowing attention to be directed to various spatial or non-spatial features of a source. However, we know little about how much selective attention processes are conserved across sensory modalities, or how different areas of the brain are differentially engaged depending on the feature being attended.

In vision, where sensory acuity changes with eccentricity from the fovea, spatial attention, and eye gaze circuitry are intimately intertwined (Corbetta et al., 2008). The frontal eye fields (FEFs), located in premotor cortex, both control eye gaze and participate in the cortical network that directs spatial attention even without eye movement (Bruce et al., 1985; Wardak et al., 2006). In audition, where gaze direction does not alter the acoustic information reaching the ears, the relationship between eye gaze and selective attention is less clear (Gherri et al., 2008). Nonetheless, neuroimaging studies show that auditory spatial attention tasks evoke FEF activity (Mayer et al., 2006; Wu et al., 2007; Salmi et al., 2009), including when attention is directed outside the visual field of view (Tark and Curtis, 2009). Moreover, saccade preparation and gaze direction can affect performance in audiospatial tasks (Pavani et al., 2005).

Past visual studies suggest that a frontoparietal network participates in top-down control of attention to both spatial and non-spatial features (Giesbrecht et al., 2003; Slagter et al., 2007). However, non-spatial attention engages additional areas beyond those involved in spatial attention. For instance, prior to visual stimulus onset, attention to color increases activity in a color-responsive region in occipital cortex, suggesting modulatory control in anticipation of upcoming stimuli (Slagter et al., 2007). In a study involving shifting of attention between auditory and visual stimuli, Shomstein and Yantis (2006) observed distributed activation including bilateral parietal lobule [and superior temporal sulcus (STS)], consistent with the idea that audition and vision share a common supramodal attention network. Here, we contrast whole-brain activity during spatial and non-spatial auditory attention tasks, asking whether different cortical areas participate when listeners attend to location versus pitch. Given the involvement of the FEFs in visual spatial attention, gaze control, and orientation, we hypothesized that when preparing to attend to an upcoming sound, FEFs would be engaged more strongly when attention was directed to spatial location than to pitch.

Multiple previous studies have contrasted neural activity for tasks in which subjects either judged spatial location or judged pitch in order to understand how these different features are encoded. When listeners judged sequences where either location or pitch changed from token to token, differences in cortical activity were found at the level of planum temporale and Heschl’s gyrus (Warren and Griffiths, 2003). Left and right premotors areas show different levels of fMRI response when localizing auditory stimuli compared to when recognizing stimuli (Maeder et al., 2001). Another study showed that bilateral premotor areas (likely including FEFs) are more strongly activated when attending to stimuli based on space versus pitch differences, whereas bilateral superior temporal areas are active in both tasks (Degerman et al., 2006). While all of these studies point to areas that are preferentially involved in processing location over pitch (or vice versa), in each of these studies, the stimuli changed along with the task demands. Thus, activity differences could be the result of bottom-up stimulus differences as well as task demands; results of these studies cannot disentangle which activity differences are due purely to differences in type and distribution of input stimuli and which are due to differences in attentional focus.

A smaller set of studies have utilized identical auditory stimuli while manipulating what aspect of the stimulus is judged or which competing sound a listener must attend to perform a task. One PET study found that right premotor areas (possibly including right FEF) are involved when auditory attention is based on either the location or the pitch of tone sequences (Zatorre et al., 1999). Ahveninen et al. (2006) found evidence for “what” and “where” pathways in the STS, as shown by adaptation to repeated presentation of stimuli, with sharper spatial tuning in posterior STS and sharper phoneme tuning in anterior STS. Although these studies implicate premotor areas and likely FEFs (along side STS and many other regions) in auditory selective attention, they do not directly differentiate between the activity evoked when a listener prepares to attend to a particular auditory stimulus versus the activity evoked during auditory object selection (when the attended stimulus is being presented).

One previous fMRI study contrasting activity for attention to location and pitch found differences even on catch trials where no acoustic targets were presented, suggesting that preparatory control signals modulate neural activity in anticipation of upcoming stimuli (Hill and Miller, 2010). Here, both the inferior frontal gyrus (linked to language processing) and posterior STS showed greater activity when attention was directed to pitch versus location. Similarly, it has been shown that preparing to attend to a sound expected to originate from a given direction biases auditory cortex contralateral to that direction (Voisin et al., 2006). These studies indicate that preparing to attend in the auditory modality engages a distributed cortical network. However, it remains unclear the extent to which the FEFs are involved in such preparatory activity (and how this differs depending on whether the subject prepares to attend based on spatial location or some non-spatial feature, such as pitch), despite their previously observed involvement in auditory attention.

Moreover, although these kinds of fMRI and PET designs may tease apart neural activity linked purely to attentional control (i.e., before the to-be-attended stimulus onset) versus changes in responsiveness to particular sensory inputs (i.e., during the stimulus), the long time scale of the BOLD response obscures rapid cortical dynamics. Moreover, while source localization in anatomically constrained magnetoencephalography (MEG) studies is not as precise as fMRI, MEG also allows us to measure neuronal currents directly instead of indirectly via the fMRI BOLD signal. One early event-related potential study, for example, observed scalp potentials spanning 80–700 ms following auditory stimuli containing to-be-attended spatial or frequency characteristics (Woods et al., 1994), although the neural generators of such potentials remain unclear.

Here, we asked subjects to report a spoken target digit from a mixture of two simultaneous digits while we measured MEG signals. Anatomical information from MRI scans constrained estimates of the sources of neural activity. By trading some of the spatial precision of fMRI (for comparison, see Sharon et al., 2007) for the millisecond time resolution of MEG, this approach allowed us to determine the cortical regions engaged during auditory attention direction (in the pre-stimulus preparatory period) as well as object selection (in the stimulus period) with fine temporal precision.

## Materials and Methods

### Subjects

Seventeen normal-hearing subjects participated in the experiment (18–35 years of age, two females. This unequal distribution occurred by chance; however, we do not expect the generalizability of our findings to be affected by the inclusion of only two females as, to our knowledge, there is no evidence for sex differences in attention networks). Each gave informed consent approved by Massachusetts General Hospital and Boston University. All participants had clinically normal-hearing (bilateral thresholds within 20 dB of normal-hearing thresholds). Since the MEG room is magnetically but not acoustically shielded, continuous, diffuse white noise (inverted at one ear to generate interaural differences that cause the noise to “fill the head,” rather than coming from a distinct location) was presented at 60 dB SPL throughout the experiment to mask any environmental sounds during MEG acquisition. The token-to-noise ratio was 20 dB, ensuring that all speech stimuli were heard easily and were intelligible.

### Stimuli

Visual stimuli (left, right, up, and down cue arrows; response circle; and fixation dot) were presented using PsychToolbox (Brainard, 1997) and a Digital Light Processing InFocus 350 projector (Texas Instruments) onto a back-projection screen placed 1 m in front of participants. Auditory tokens consisted of the spoken digits 1–4 (average duration of ∼400 ms) from the TIDIGIT database (Leonard et al., 1984). Digits were sampled at 24.4 kHz and windowed by 10-ms-long squared-cosine rise/fall ramps. The pitch of each token was monotonized using Praat (Boersma and Weenink, 2012); the high- and low-pitch stimuli were generated at 100 Hz ± 3 semitones (=119 and 84 Hz), respectively. Tokens were processed by non-individualized head-related transfer functions to simulate sources 30° to the left or right of midline (with HRTFs sourced from Shinn-Cunningham et al., 2005). Sound stimuli were presented using Tucker-Davis Technologies hardware (RP2.1 and HB7) and Tubal Insertion Earphones (Nicolet Biomedical Instruments, WI, USA; model TIP-300 300 Ω).

### Task

**Figure 1:**
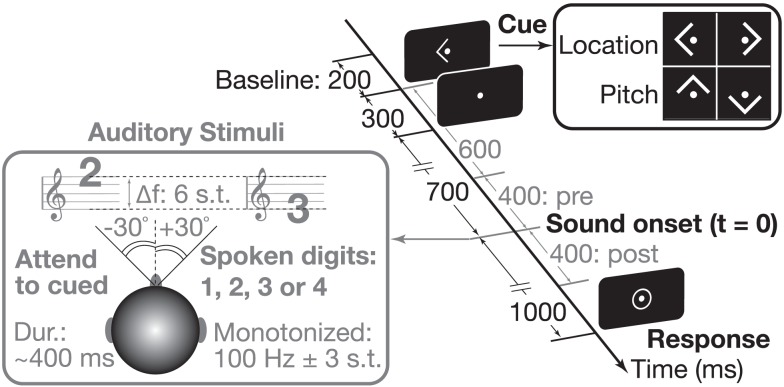
**We used an auditory attention paradigm that visually cued subjects to attend to either the location (left/right) or the pitch (up/down) of an upcoming sound**. Throughout each trial, subjects were asked to maintain fixation on a center dot (0.3° visual angle). A 300-ms-long arrow cue (1.0° visual angle) instructed subject what to attend in the upcoming sound (see right inset). The sound mixture, presented 700 ms after the arrow cue was extinguished, consisted of two spoken digit tokens (see left inset). The pitch of the speech was manipulated using Praat (Boersma and Weenink, 2012); spatial location was controlled by processing with head-related transfer functions. Listeners reported the target digit (values 1–4) by a button press after the appearance of a center ring.

Each subject performed 288 trials presented in a pseudorandom order, broken up into four runs each consisting of 72 trials (lasting ∼5 min). Subjects were instructed to maintain gaze fixation on a dot at the center of the screen throughout each run. At the beginning of each trial, subjects were cued by an arrow (300 ms duration) to attend to one of two simultaneous spoken digits (that began playing 700 ms after the visual cue ended) based on either the target pitch (up/down arrows for high/low pitches) or the target spatial location (left/right arrows). On each trial, the target digit had a given pitch and location (e.g., 119 Hz from the left), and the masker had the complementary attributes (e.g., 84 Hz and from the right). Subjects were to attend to the cued stimulus and determine the spoken digit. One second after sound onset (with digit duration ∼400 ms) a response circle appeared to indicate that subjects should identify the target digit (defined by pitch or location) by pushing the appropriate button on a four-button response box using their right hand. Motor artifacts were minimized by having listeners respond at the end of each trial, as cued by a center circle (Figure 1). Since subjects were instructed to respond at any point during this response period and the delay between digit onset and response period was sufficiently long (1 s), we did not measure reaction times in this task. The response circle remained visible for 1 s, and ∼1 s after it disappeared (leaving the fixation dot showing) the next trial began. Across trials, stimulus spatial locations and pitches were independently randomized and counter-balanced; each trial contained one high-pitch and one low-pitch digit, with the high-pitch stimulus coming from either the left or right side and the low-pitch stimulus coming from the opposite hemifield. Thus attend-pitch and attend-space trials had identical acoustical conditions, differing only in terms of what feature the subject was instructed to attend (pitch or location). Subjects performed four behavioral runs, each lasting roughly 5 min. Cued pitch (up/down) and location (left/right) trials were randomly intermingled, counter-balanced within each run. Prior to starting these experimental runs, subjects practiced the task and were trained to respond at the appropriate time.

### MEG data acquisition

Magnetoencephalography data were acquired inside a magnetically shielded room (IMEDCO) using a MEG (306-channel dc-SQUID Neuromag VectorView system (Elekta-Neuromag) with 204 planar gradiometers and 102 axial magnetometers. Two bipolar electro-oculogram (EOG) electrode pairs measured eye movements and blinks. The data were recorded at a sampling rate of 600 Hz with a bandpass of 0.1–200 Hz. Four head position indicator (HPI) coils were used to monitor head position (see Liu et al., 2010 for detailed description). At the beginning of each run, magnetic fields from the HPI coils were recorded to calculate the position and orientation of the head relative to the MEG sensor array.

### MEG data processing

All channels were processed using the signal-space separation method (Taulu et al., 2005), which suppresses environmental noise. In addition, we eliminated the subspaces containing heartbeats and blinks using the signal-space projection method (Uusitalo and Ilmoniemi, 1997), which uses the spatial covariance (across channels) during automatically identified (MATLAB software) epochs containing heartbeats or blinks to remove artifacts due to these physiological processes (e.g., see Lee et al., 2012). The waveforms for each trial type were then averaged for each subject. During offline averaging (bandpass 0.1–55 Hz), trials were rejected if the behavioral response was incorrect, if eye movements or blinks were recorded (EOG greater than 200 μV), or if MEG exceeded either 10 pT in magnetometers or 3 pT/cm in gradiometers. Signal-space projection of blink artifacts was used in conjunction with epoch rejection to conservatively remove any residual effects of blink/saccade generation in our data not addressed by epoch rejection.

Magnetoencephalography data were analyzed with a whole-brain analysis using the MNE software suite (http://www.nmr.mgh.harvard.edu/mne). A noise-covariance matrix was estimated from the 200-ms baseline periods prior to the onset of each trial (before the visual cue). The anatomically constrained linear minimum-norm estimate was used to compute dipole amplitudes at each cortical location (for details, see Lee et al., 2012), which were temporally averaged using 50 ms non-overlapping windows. Because subject head positions were consistent across runs, we averaged the forward solutions from each run (incorporating the head positions) before calculating the inverse operator. This allowed us to use a single inverse per subject. For pre- and post-sound stimulus analysis, activity was measured during 400-ms epochs prior to and after sound onset (preparatory and stimulus periods) following a baseline correction calculated from the 200-ms period prior to the visual cue (up/down/left/right) that signaled the start of each trial. For across-subject comparisons, source localized data were morphed to a template brain, optimally aligning individual sulcal-gyral patterns (Fischl et al., 1999).

### Statistical analysis

For displaying group-level activity on the cortical surface, we first spatially smoothed individual subject data across neighboring vertices. Specifically, for 25 successive iterations of the spreading operator, the new value at each vertex was the sum of the previous values of the vertex and its immediate neighbors (adjacent vertices in the parcellation of the cortical surface), divided by the number of non-zero values included. This smoothing helps to compensate for expected anatomical and functional subject differences. The smoothed estimates were used to compute a location versus pitch contrast at each vertex on the cortical surface. The resulting estimates were submitted to a repeated-measures ANOVA, treating time as an experimental factor over eight consecutive 50-ms time frames making up both the preparatory and stimulus epochs. A conservative Greenhouse–Geisser non-sphericity correction was used within each 400-ms epoch to mitigate the effect of possible correlations of the residuals over the time bins. To compensate for multiple-comparisons, we took the conservative approach of displaying *p*-values on the cortical maps after a stringent Bonferroni correction taking into account the number of source vertices across the brain (∼10,000/hemisphere). Although a given brain area may be activated strongly in both the attend-location and attend-pitch conditions, this within-subjects analysis only measures the differences in activation elicited during the two tasks. This analysis ignores the overall activation levels in the conditions to reduce the potential for biases in our measurements to affect results. Given this, we only discuss the differential activity across the two tasks. To examine large regions of activity, we only examine clusters containing at least 100 vertices.

### Saccade paradigm and eye movement processing

We used 192 trials of a memory-guided go/no-go saccade task to obtain an FEF functional localizer. Each saccadic trial lasted 3.3 s (counter-balanced for left/right movement). A left or right arrow (300 ms in duration) began each trial, after which a 1-s-long ring was presented, shifted to the side by 10° in the direction of the preceding arrow cue. Subjects were asked to maintain center fixation (white dot subtending 0.3°) unless otherwise instructed. The color of the arrow on a given trial cued the subjects either to move the eyes (“go”; green) or to keep eyes fixed (“no-go”; red). On a separate block, we asked subjects to track a white dot (subtended visual angle, 0.3°) with their gaze as it moved to one of six locations (± 3°, 6°, or 9° from center, each presented once in random order within a run, with four runs making up the block). These tracking data were used to obtain an individualized linear transform relating EOG to eye gaze eccentricity in degrees. Saccades were identified as horizontal eye movements with velocities exceeding 100°/s. The onset of a saccade was defined as the point at which the velocity of the eye first exceeded 30°/s. Trials with saccade latencies less than 100 ms were considered anticipatory and were not included in subsequent analysis.

### Additional saccade monitoring

In order to rule out eye saccade explanations of results in our spatial auditory attention task, one subject was invited back to perform a full auditory attention session in the MEG environment while eye gaze was monitored using high-resolution binocular eye-tracking. Real-time (1000 Hz sampling) binocular gaze position was determined using an Eyelink 1000 MEG compatible eye-tracking system (SR Research, Ltd., ON, Canada) calibrated using a 9-point fixation paradigm at the beginning of each block and drift corrected at the beginning of each trial. This system has a spatial resolution of 0.02° (RMS) and accuracy of (average bias up to) 0.25°. We used eye-tracking data from this system both to determine if there were significant differences between the eye positions in attend-space and attend-pitch conditions, and to test whether there was a correlation between eye position and FEF activation on a single-trial level. Since it is possible that single-trial MEG activations may be too noisy to provide a meaningful comparison between conditions, we also compared trial-averaged FEF activations and EOG levels across subjects. To remove effects due to bias (e.g., variation in overall EOG or FEF amplitudes across subjects), we compared EOG to FEF activations using the normalized differences of each in the space and pitch conditions as (space − pitch)/(space + pitch). Additionally, to deal with the possibility that left and right trials could have opposing eye-direction movements that would cancel out in averaging, we compared LFEF activation to the mean of the (1) magnitude of the left-trial-averaged EOG and (2) magnitude of the right-trial-averaged EOG.

### FEF-ROI functional localizer

*A priori*, we focused only on the FEFs, located in and around the precentral sulcus and gyrus (Simó et al., 2005). For each subject, we used a functional localizer to obtain a region of interest (ROI) anatomically constrained to the bilateral superior and inferior precentral sulci and the precentral gyri, as defined by an automated surface-based parcellation (Fischl et al., 2004). Within these regions in the averaged group data, we functionally constrained the FEF-ROI to vertices showing activity (i.e., differences in dipole strengths) in the “go” versus “no-go” saccade contrast with a threshold of *p* < 0.05 following a conservative Greenhouse–Geisser non-sphericity correction. For this analysis, activity was estimated every 50 ms between 0 and 300 ms after the onset of the peripheral ring. This contrast between the “go” and “no-go” trials isolates saccade-generating signals associated with the FEFs. This provided cross-subject spatial localization data for the FEFs to compare to our findings from the whole-brain analysis.

## Results

### Left-dominant differential engagement in auditory spatial and pitch attention

**Figure 2:**
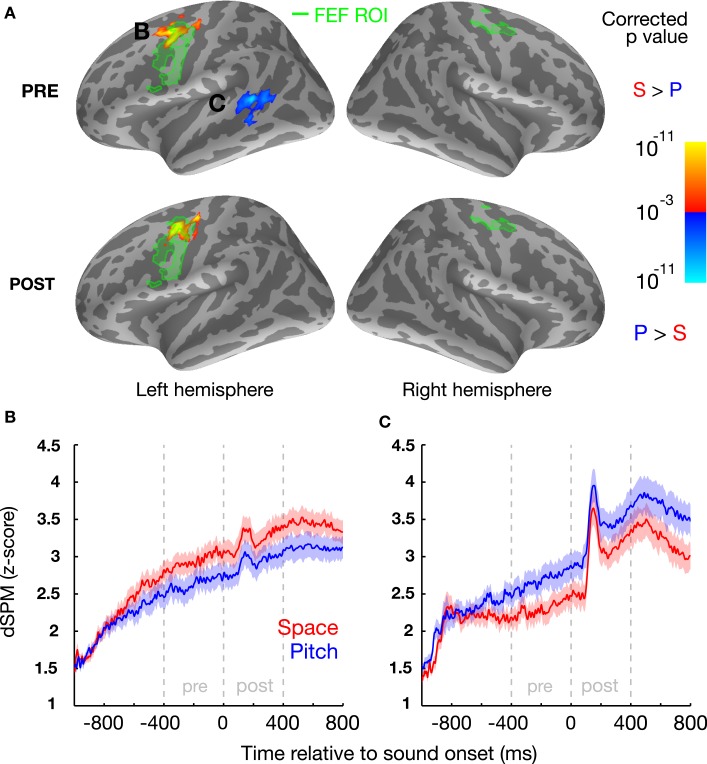
**Left FEF and left STG are more active prior to sound onset when subjects attend to space and pitch, respectively, based on contrasts of the cortical signal in the two conditions**. **(A)** Statistical map (group average) displayed on the inflated cortical surface of the left and right hemispheres, illustrating a vertex-by-vertex comparison (yellow: greater activity in location trials; blue: greater activity in pitch trials; minimum cluster-size threshold 100 vertices). Functionally localized FEF regions are highlighted in green. B/C: normalized evoked cortical current time courses for the significant left FEF **(B)** and left STG regions **(C)** for both location and pitch conditions, shown with standard error bars (μ ± SEM) across subjects.

To quantify the cortical involvement in top-down attention, we analyzed the differential cortical source estimates between location and pitch trials in the 400-ms-long preparatory (from 600 ms after the onset of the visual cue directing attention up to sound onset) and stimulus epochs (from sound onset to 400 ms later; note that the stimulus epoch analysis window encapsulates the duration of each token; see Figure 1). We also located our primary *a priori* ROI, FEFs, based on a combination of anatomical landmarks (limiting analysis to the precentral sulcus and gyrus; see Materials and Methods) and significant functional activity from our memory-guided, go/no-go saccade task, which revealed a larger area of activation for in the left hemisphere than the right (Figure 2A, green labels; left/right ROI center of mass *x* = −47.5, *y* = −1.8, *z* = 39.6, and *x* = 34.8, y = −9.0, z = 52.8, respectively; activation traces shown in Figure 2B). This may reflect the hemispheric asymmetry related to the functional localizer task used, consistent with recent findings that the oculomotor system is more asymmetric in humans than in monkeys (Kagan et al., 2010).

A region in the left, but not right, dorsal precentral sulcus/gyrus was more active when subjects attended to location than when they attended to pitch (Figure 2A), both in the preparatory and stimulus epochs. Importantly, this region of enhanced activity during spatial attention (MNI centroid coordinates: *x* = −36.3, *y* = −4.8, *z* = 42.4) overlapped with the left FEF-ROI. We also found that the left, but not right, posterior STS (MNI centroid coordinates: *x* = −49.7, −45.1, 7.1) was more active when subjects attended to pitch than when they attended to location, but only in the preparatory epoch.

Electro-oculogram results revealed no systematic differences in eye movements for the location and pitch trials. For the subject in whom eye movements were recorded, there was no evidence for any consistent directional bias in either eye position or gaze velocity that depended on trial type, either when treated as a function of peristimulus time or when using the ergodic average (*p* > 0.2 for all paired *t*-test comparisons; left, right, and pitch trial types). In the attend-pitch trials, we found no correlation between eye position and the activation in the LFEF region that was significantly more active during attend-space than attend-pitch trials (Kendall *p* = 0.308 for both vertical and horizontal position, *N* = 116). In addition, following a multiple-comparisons threshold correction (α = 0.05/4 = 0.0125), the correlation between horizontal eye position and FEF activation on pitch trials was also not statistically significant (Kendall *p* = 0.413, *N* = 68); importantly, even if this difference was found to be statistically significant, it cannot explain why LFEF activation was greater in space trials than in pitch trials. Moreover, when examining LFEF across all subjects, the normalized trial-averaged activation differences in LFEF did not correlate with either the horizontal or vertical (Kendall *p* > 0.73 for both, *N* = 17) EOG. These data all suggest that the differential FEF engagement was not simply due to eye movements in “attend left” and “attend right” trials. We also compared bilateral “attend left” and “attend right” activity, and found no significant difference in activity in either left or right FEF with direction of spatial attention (data not shown).

### Behavioral results and relationship to neural activity

Overall, subjects performed the task accurately in both location and pitch trials, but were slightly better on location trials (percent correct 86.42 ± 3.27%, mean ± SEM) than on pitch trials (78.32 ± 3.97%; *P* = 0.0002, paired *t*-test). All subjects performed better than chance (25% correct), although performance varied substantially across subjects, from 56.3 to 99.3% when attending location and from 46.5 to 99.3% when attending pitch. Any trials in which a valid response was not recorded during the fixed response period were designated misses. In general, misses represented a small fraction of trials: 1.3–8.9% for all but three subjects, who had miss rates of 13.9–25.0%. For the three subjects with higher miss rates, most misses arose because responses were made before the response period (i.e., the subjects performed the task and responded, but did so too quickly). If responses up to 0.4 s before the onset of the response circle (i.e., at least 200 ms following sound offset) were included, the miss rates for all three of these subjects would decrease to 1.4–5.2%. To ensure that motor responses did not interfere with the results presented here, these anticipatory responses were not included in our MEG analysis.

Percent correct performance was correlated in attend-location and attend-pitch trials across subjects (Kendall tau = 0.857, *P* < 0.000003, *N* = 17), showing that some of the individual differences were unrelated to what feature was attended, coming instead from individual differences in the ability to focus auditory selective attention and perform the task. Similarly, the degree of modulation in left FEF activity for attend-location versus attend-pitch trials was negatively correlated with the degree of attended-feature-specific modulation of activity in left STS (Kendall tau = −0.467, *P* < 0.011, *N* = 17), consistent with there being a common attentional control signal that affects activity in these areas in a manner specific to the feature to which attention is directed. However, comparing average performance on the two tasks to the average of the modulation in left FEF and the negative of the modulation in left STS, there was no significant relationship (Kendall tau = −0.081, *P* = 0.680, *N* = 17). Comparing the level of neural activation (relative to baseline) in the space condition with the activation in left FEF, and in the pitch condition with left STS, correlations were insignificant but trending (Kendall tau = 0.32 and, *P* = 0.082 and 0.069, respectively, *N* = 17). Although left FEF activation in the pitch condition was correlated with performance on the pitch task (Kendall tau = 0.41, *P* = 0.026, *N* = 17), after Bonferroni correction for six comparisons (adjusted α = 0.0083), this difference was not statistically significant. Activity in the left STS was not significantly correlated with performance on the space condition (Kendall tau = 0.31, *P* = 0.097, *N* = 17).

## Discussion

Our results demonstrate that regions of the cortex are engaged in directing attention to acoustic features even before the sounds begin; moreover, different regions are engaged more strongly depending on what feature is directly selective auditory attention: left FEF when attending location and left posterior STS when attending pitch. Previous fMRI studies demonstrate that activity in a left dominated frontoparietal network is enhanced during attentionally demanding trials compared to fixation trials, whether subjects attend to a spatial or a non-spatial feature, and in both visual and auditory tasks. During a visual attention task, anatomical regions proximal to bilateral FEFs were more active during spatial attention, while the left ventral occipital cortex was more active when subjects attended to color (Giesbrecht et al., 2003; Slagter et al., 2007). During an auditory task, left FEF showed enhanced activity for both location and pitch, even on catch trials where there was no acoustic stimulus (Hill and Miller, 2010), consistent with the anticipatory activity we found in our task. However, in this earlier study, right FEF was more active in location trials, and the inferior frontal gyrus (a region linked to language processing) was more active in pitch trials. While it is difficult to directly compare results of studies using different sensory stimuli and different neuroimaging techniques, especially since the relationship between neural activity measured using MEG and BOLD responses measured using fMRI is not well established, our results add to evidence that FEFs are involved in control of covert spatial attention across different modalities (while other areas may be similarly engaged when attending to non-spatial features). In contrast to previous studies, our results suggest that there is an asymmetry in auditory processing whereby left FEF is more strongly involved in attending to auditory stimuli based on spatial location compared to pitch. We also show that this attention-specific control begins in preparation for upcoming stimuli containing a to-be-attended feature. It is worth noting that the activity observed in preparing to attend to stimuli based on spatial condition may be due to the deployment of both auditory and visual attention to the spatial location of interest, as would likely be the case if auditory and visual spatial attention share a common supramodal network. Teasing this apart could be interesting in future studies that make use of either auditory-only cues or visual cues that come on well before auditory attention must be directed. However, our observations here are unlikely to be due solely to the deployment of visual attention.

It has been shown previously that preparing to attend to a sound likely to originate from a given direction biases cortical activity in auditory cortex contralateral to that direction (Voisin et al., 2006), indicating that prior to sound onset, listeners “prime” cortical representations to favor upcoming sounds from the direction to be attended. Given this, our results are consistent with our listeners engaging auditory attention to perform our tasks, although we cannot rule out the possibility that listeners co-deploy both auditory and visual spatial attention networks in anticipation of an upcoming sound. Additionally, although some evoked responses are visible in the traces of left FEF and STS (likely due to leakage from primary sensory cortices), the observed differences reported here must be due to the task condition (attend-space versus attend-pitch) since the acoustic stimuli used in the two conditions are identical.

The left lateralization of FEF activity initially seems at odds with past reports of “hemispheric dominance.” It is well established that the right hemisphere processes information in both visual fields, whereas the left hemisphere exclusively encodes the right visual field (Mesulam, 1981). This raises the question of why left, but not right, FEF is more active in our location trials, regardless of the direction of the target, and why there are no significant differences in FEF activity for “attend left” versus “attend right” trials. It is unlikely that this is due to preparatory motor activation (i.e., preparing to press a button with the right hand), since such activity would be the same for both the space task and the pitch task that were contrasted, yet differences in activation were observed before a sound stimulus was presented (and thus before an appropriate response could be prepared). We believe this left FEF bias may reflect its participation in a dorsal, top-down attention network (Corbetta et al., 2008), with right FEF involved in top-down attention, exogenous attention, and shifting of attention. Previous auditory fMRI studies that find bilateral FEF activation during auditory spatial attention tasks used paradigms that differ from ours: most either required subjects to explicitly shift their auditory spatial attention (Salmi et al., 2007, 2009), or exogenously cued the auditory location to attend (Wu et al., 2007). Moreover, because of scanner noise, listeners in these studies may have deployed some form of non-spatial attention to focus on the desired acoustic stimuli instead of or in addition to engaging spatial attention. The one study that required top-down deployment of auditory spatial attention (Hill and Miller, 2010) yielded poor behavioral performance (especially early in the experiment), suggesting that the subjects were not always successful in deploying attention, and sometimes reoriented attention while trying to perform the task. In contrast, our study presented a brief stimulus (one syllable long), yet subjects performed the task reasonably well, showing that they successfully deployed top-down spatial attention in the preparatory period. Thus, we suggest that the left FEF is differentially more involved in top-down auditory spatial attention, consistent with the supramodal attentional network previously proposed (Corbetta et al., 2008). Note that although left FEF shows greater activity during spatial rather than pitch-based attention trials, left FEF also may well play a role in non-spatial attention as well, as evidenced by a significant (before a multiple-comparisons correction) correlation between activity in left FEF and behavioral performance on attend-pitch trials. The pre-auditory-stimulus left FEF activity observed here is also similar to the anticipatory activity in FEFs reported in past visual studies that is linked to top-down control of spatial attention (Awh et al., 2006).

We found that left posterior STS, which was not chosen *a priori* as an ROI, was recruited in attend-pitch trials during the preparatory period, a result that mirrors previous findings of other cortical regions showing attention biases for non-spatial features: ventral occipital cortex for attention to color (Giesbrecht et al., 2003, 2006; Slagter et al., 2007), inferior frontal regions for attention to spectral features in a language-related task (Hill and Miller, 2010), and preparatory activity in auditory cortex contralateral to the expected location of an upcoming sound (Voisin et al., 2006). Several studies have associated the left STS with the identification or categorization of sounds based on non-spatial attributes (Möttönen et al., 2006; Liebenthal et al., 2010), especially for people with absolute pitch (Schulze et al., 2009). Although this area is likely involved in performing categorization in both the spatial location and pitch tasks, our results suggest that the cortical region associated with categorization of pitch information becomes more active and helps listeners prepare to select a target stimulus based on pitch. These results support the existence of different pathways for processing “what” and “where” sound attributes (e.g., Rauschecker and Tian, 2000; Ahveninen et al., 2006); however, since the posterior location of the left STS activation observed here is more consistent with the previously reported “where” pathway, additional experiments will be necessary to provide the spatial resolution required to definitively tease apart the contributions of these areas. Moreover, we were relatively conservative in our data analysis (e.g., Bonferroni correction) to decrease the likelihood of false positives; however, this increases the chance that additional areas are significantly involved during tasks like those used here; experiments that relax constraints or that employ other statistical approaches might expose such other activity (e.g., Singh et al., 2003; Maris and Oostenveld, 2007). For example, the use of masking noise could obscure differences in activity that the two tasks might have evoked in auditory cortex for presentations in quiet, especially given the conservative analyses we adopted. It is also possible that there is an underlying activity difference in left STS during the stimulus (“post” period; as seen in Figure 2C) as well; future studies with better SNR or less strict thresholding may well observe significant left-biased activity differences during the stimuli when attending based on pitch.

Finally, our results show that left FEF is involved both before and after the onset of sound while activity in left posterior STS is significantly enhanced only prior to the onset of sound. These changes were correlated across subjects, as if the degree of attentional modulation in both attend-location and attend-pitch trials depends on some common signal, regardless of the feature attended. However, these activity differences are not significantly correlated with behavioral performance, even though listener ability varies widely across subjects. In our neural analysis, we contrasted two conditions, each of which engages selective auditory attention; thus, any differences in the strength with which listeners engage cortical regions that are common to both attend-location and attend-pitch trials is invisible in our analysis; we only see the indirect effects of such common control in the strength of modulation of the feature-specific areas left FEF and left STS. Combined with the observation that across subjects, performance is strongly correlated in the attend-location and attend-pitch trials, our results suggest that overall selective attention performance depends on the degree of engagement of neural areas that are employed both when attending to location and when attending to pitch, and/or on individual differences in the fidelity of sensory encoding of the basic acoustic information needed to compute auditory features like location and pitch (Ruggles et al., 2011). Here, we also found insignificant but trending correlations between activity in attend-space and attend-pitch trials. However, the estimates of neural activity normalized to baseline used here are influenced by the signal-to-noise ratio and the number of valid trials for each subject, which could contribute to the lack of observed significant correlations. Future experiments thus could be undertaken to explore the degree to which the overall activity of a general “attention network” helps to predict individual ability on this kind of selective auditory attention task. Additionally, although we did not have a sufficient number of incorrect trials to perform a meaningful analysis here, future studies could also look at activations in error trials to also examine the auditory selective attention network.

Taken in the context of previous psychoacoustical and neuroimaging work, our findings support the conclusions that (1) left FEF is involved in both directing and sustaining auditory spatial attention and (2) the left STS aids object selection based on its pitch feature prior to the onset of sound.

## Conflict of Interest Statement

The authors declare that the research was conducted in the absence of any commercial or financial relationships that could be construed as a potential conflict of interest.
